# Prognosis Stratification Tools in Early-Stage Endometrial Cancer: Could We Improve Their Accuracy?

**DOI:** 10.3390/cancers14040912

**Published:** 2022-02-12

**Authors:** Jorge Luis Ramon-Patino, Ignacio Ruz-Caracuel, Victoria Heredia-Soto, Luis Eduardo Garcia de la Calle, Bulat Zagidullin, Yinyin Wang, Alberto Berjon, Alvaro Lopez-Janeiro, Maria Miguel, Javier Escudero, Alejandro Gallego, Beatriz Castelo, Laura Yebenes, Alicia Hernandez, Jaime Feliu, Alberto Pelaez-García, Jing Tang, David Hardisson, Marta Mendiola, Andres Redondo

**Affiliations:** 1 Department of Medical Oncology, Hospital Universitario La Paz, 28046 Madrid, Spain; georgeram207@gmail.com (J.L.R.-P.); luiseduardo.garcia@salud.madrid.org (L.E.G.d.l.C.); alejandro.gallego@salud.madrid.org (A.G.); beatriz.castelo@salud.madrid.org (B.C.); jaime.feliu@salud.madrid.org (J.F.); 2 Department of Pathology, Hospital Universitario La Paz, 28046 Madrid, Spain; ignacio.ruz@salud.madrid.org (I.R.-C.); alberto.berjon@salud.madrid.org (A.B.); aljaneiro@salud.madrid.org (A.L.-J.); laura.yebenes@salud.madrid.org (L.Y.); david.hardisson@salud.madrid.org (D.H.); 3 Translational Oncology Research Laboratory, Hospital La Paz Institute for Health Research (IdiPAZ), 28046 Madrid, Spain; victoriam.heredia@salud.madrid.org (V.H.-S.); maria.miguel@dipaz.es (M.M.); fjescudero@isciii.es (J.E.); 4 Center for Biomedical Research in the Cancer Network (Centro de Investigación Biomédica en Red de Cáncer, CIBERONC), Instituto de Salud Carlos III, 28029 Madrid, Spain; 5 Research Program in Systems Oncology, Faculty of Medicine, University of Helsinki, 00290 Helsinki, Finland; bulat.zagidullin@helsinki.fi (B.Z.); yinyin.wang@helsinki.fi (Y.W.); jing.tang@helsinki.fi (J.T.); 6 Molecular Pathology and Therapeutic Targets Group, Hospital La Paz Institute for Health Research (IdiPAZ), 28046 Madrid, Spain; alberto.pelaez@idipaz.es; 7 Cátedra UAM-ANGEM, Faculty of Medicine, Universidad Autónoma de Madrid, 28029 Madrid, Spain; 8 Department of Obstetrics & Gynaecology, Hospital Universitario La Paz, IdiPAZ, 28046 Madrid, Spain; ahernandezg@salud.madrid.org; 9 Faculty of Medicine, Universidad Autónoma de Madrid, 28029 Madrid, Spain

**Keywords:** endometrial cancer, prognosis, risk assessment, biomarkers, *CTNNB1*

## Abstract

**Simple Summary:**

Endometrial cancer is the most common gynaecological malignancy in developed countries. Most cases are diagnosed at a localized stage, overall with a good prognosis, although approximately 15% of them will recur. The identification of patients with an increased risk of relapse remains a challenge for clinicians. There are well-defined clinicopathological characteristics associated with prognosis. These variables have been integrated in multiple classifiers to stratify the prognosis, and more recently, molecular features have also been considered. The aim of our retrospective study was to compare the three available prognostic stratification tools for endometrial cancer and determine if additional biomarkers could improve their accuracy. We confirmed that the incorporation of molecular classification in risk stratification resulted in better discriminatory capability, which was improved even further with the addition of *CTNNB1* mutational evaluation.

**Abstract:**

There are three prognostic stratification tools used for endometrial cancer: ESMO-ESGO-ESTRO 2016, ProMisE, and ESGO-ESTRO-ESP 2020. However, these methods are not sufficiently accurate to address prognosis. The aim of this study was to investigate whether the integration of molecular classification and other biomarkers could be used to improve the prognosis stratification in early-stage endometrial cancer. Relapse-free and overall survival of each classifier were analyzed, and the c-index was employed to assess accuracy. Other biomarkers were explored to improve the precision of risk classifiers. We analyzed 293 patients. A comparison between the three classifiers showed an improved accuracy in ESGO-ESTRO-ESP 2020 when RFS was evaluated (c-index = 0.78), although we did not find broad differences between intermediate prognostic groups. Prognosis of these patients was better stratified with the incorporation of *CTNNB1* status to the 2020 classifier (c-index 0.81), with statistically significant and clinically relevant differences in 5-year RFS: 93.9% for low risk, 79.1% for intermediate merged group/*CTNNB1* wild type, and 42.7% for high risk (including patients with *CTNNB1* mutation). The incorporation of molecular classification in risk stratification resulted in better discriminatory capability, which could be improved even further with the addition of *CTNNB1* mutational evaluation.

## 1. Introduction

Endometrial cancer (EC) is the most common gynaecological malignancy in developed countries. Most cases are diagnosed at a localised stage, reaching 5-year survival rates of over 95% in some series [1,2]. Despite such a good prognosis, approximately 15% of patients with early stages (I and II) of EC will recur [3]. Therefore, the identification of patients with an increased risk of relapse remains a challenge for clinicians.

There are well-defined characteristics associated with prognosis, including age, lympho-vascular space invasion (LVSI), myometrial infiltration, differentiation grade and International Federation of Gynecology and Obstetrics (FIGO) stage [4]. During the past 2 decades, these variables were integrated in multiple classifiers to stratify the prognosis. In 2016, the European Society of Medical Oncology (ESMO)-European Society of Gynecologic Oncology (ESGO)-European Society for Radiotherapy and Oncology (ESTRO) Consensus established a four-group classification (low, intermediate, high-intermediate and high risk) based on clinicopathological features, with the aim of prognosis stratification, but also to help with the indication for adjuvant therapy [2].

The Tumor Cancer Genome Atlas (TCGA) performed a comprehensive genomic profiling of over 300 EC samples, resulting in a molecular classification with prognosis implications [5]. In terms of a more cost effective and applicable method for group assignment in routine practice, the Leiden/PORTEC and the Vancouver/Proactive Molecular Risk Classifier for Endometrial Cancer (ProMisE) groups reproduced the TCGA molecular classification using surrogate biomarkers by targeted sequencing and immunohistochemistry (IHC) on formalin-fixed paraffin-embedded (FFPE) tumour samples [1,6,7,8]. The group named POLE, composed of cases with mutations in the exonuclease domain (EDM) of polymerase-ɛ gene, has an excellent prognosis. In contrast, patients with the poorest prognosis harbour tumour mutations in the *TP53* gene. This group is named p53 abnormal (p53abn) due to aberrant immunohistochemical p53 expression. The other two groups with intermediate risk were also established. The first encompasses mismatch repair deficient (MMRd) cases, defined by loss of expression of at least one of the mismatch repair proteins (MLH1, PMS2, MSH2 and MHS6). The remaining cases are included in the group named p53 wild type (p53wt) or non-specific molecular profile (NSMP).

Furthermore, other potential prognostic biomarkers have been described in EC, although most of them remain on lab setting. For example, it is reported that oestrogen and progesterone receptors (ER and PR) play a significant role in endometrial carcinogenesis. Their expressions are associated with well-differentiated tumours and correlate with earlier tumour stages and better survival [9]. L1-cell adhesion molecule (L1CAM) overexpression has been associated with a poorer outcome [10]. Amplification and increased expression of human epidermal growth factor receptor 2 (HER2) has been correlated with poor prognosis and more aggressive tumour behaviour [11]. Those with EC harbouring catenin beta 1 (*CTNNB1)* mutation encompass a more aggressive subset within low-grade early-stage endometrioid EC [12,13]. Other biomarkers such as phosphatase and tensin homolog (PTEN), AT-rich interactive domain-containing protein 1A (ARID1A) or E-cadherin (ECAD) have also had a possible impact on prognosis in some studies [14].

The integration of clinicopathological features and molecular subgroups is currently a reality based on the recent publication of ESGO-ESTRO-European Society of Pathology (ESP) 2020 guidelines. These guidelines still recommend a four-risk group classification, incorporating ProMisE molecular markers with clinical characteristics and suggesting a possible improvement in the accuracy of the risk prognosis stratification [15].

Our aim with this study was to analyse and compare the three above-mentioned risk stratification tools in the same cohort of early-stage EC, and to identify additional biomarkers with an impact on prognosis that could improve the precision of these classifiers.

## 2. Materials and Methods

### 2.1. Study Cohort

A retrospective cohort was collected including patients diagnosed with early-stage (I and II by FIGO) EC between 2003 and 2015 at La Paz University Hospital (Madrid, Spain), with a minimum follow-up of 5 years. Patients were consecutive. The study was approved by the local Ethics Committee (HULP#PI3778) and was conducted in accordance with ethical standards of the Helsinki Declaration of the World Medical Association.

All patients underwent surgery, which consisted of a total hysterectomy and bilateral salpingo-oophorectomy. This procedure was performed initially via laparotomy, until 2006, and then by a laparoscopic approach. The lymph node assessment was performed by lymphadenectomy. We analysed clinical and pathological variables, such as age, histological subtype, FIGO stage (updating to FIGO 2009 staging system for older samples), tumour size, LVSI, grade of differentiation, and myometrial infiltration. Clinical data on treatment and follow-up were obtained from the electronic medical records database and were subsequently updated, allowing for an evaluation of relapse-free survival (RFS) and disease-specific overall survival (OS).

### 2.2. Sample Selection

Optimal tissue blocks were selected by an expert gynaecological pathologist on haematoxylin and eosin (H&E) slides. DNA was extracted from selected tumour rich regions with the Qiamp DNA FFPE Tissue Kit (Qiagen, Hilden, Germany) and used for polymerase chain reaction (PCR) purposes. Additionally, representative tumour non-necrotic areas of each case were selected for tissue microarray (TMA) construction. Two representative cores of 1.2 mm in diameter were taken and arrayed into a receptor block using a TMA workstation (Beecher Instruments, Silver Spring, MD, USA), as previously described [16].

### 2.3. Risk Stratification Tools

The ESMO-ESGO-ESTRO 2016 risk stratification groups were established as follows: low, intermediate, high-intermediate, and high risk. For simplicity, hereafter this classifier will be referred to as the ‘2016 Classifier’ [2].

We also stratified patients by the ProMisE risk groups: POLE, MMRd, p53wt/NSMP, and p53abn [7]. First, specific PCR and Sanger sequencing was performed to identify mutations in exons 9, 13 and 14 of *POLE*. These exons code for part of the EDM and account for most of the described mutations [17,18]. As a modification of the original ProMisE classification, for the POLE-mutated cases, we have only taken into account the pathogenic variants selected in the study by Leon-Castillo et al. [18]. Second, we used 4 µm sections of the TMA for IHC purposes. The expression of MLH1, PMS2, MSH2, MSH6 and p53 was evaluated with specific antibodies (p53, #IR616; MLH1, #IR079; PMS2, #IR087; MSH2, #IR084 and MSH6, #IR086 respectively), all from Agilent (Santa Clara, CA, USA), as previously described [19].

Lastly, a combination of clinicopathological and molecular variables employed in the previous risk stratification tools were used following the ESGO-ESTRO-ESP 2020 guidelines to establish a new 4-group classification: low, intermediate, high-intermediate and high risk. For simplicity. Henceforth, this will be named the ‘2020 Classifier’ [15].

### 2.4. Biomarker Analysis

Additionally, other molecular markers previously studied in EC were explored. Expressions ER, PR, ECAD, HER2, ARID1A, PTEN, and L1CAM were evaluated. Specific antibodies and cut-off categories were applied to each marker to simplify their evaluation as much as possible. A detailed description can be found in Supplementary Table S1. PCR and Sanger sequencing were also performed to explore *CTNNB1* exon 3, which contains key protein phosphorylation sites.

### 2.5. Statistical Analysis

Descriptive statistics included clinicopathological and biomarker frequencies. Qualitative variables are presented as number of cases and frequency percentages. Continuous variables are presented as median value and range. Missing values in the ProMisE and 2020 Classifier groups were imputed, taking the most frequent values from a total of 1000 runs of the predictive mean matching method provided in the mice R package [20].

The primary endpoint was to evaluate RFS, defined as the time from surgery to the time of first recurrence or death from disease. As a secondary endpoint, disease-specific OS was analysed, defined as time from the surgery to death related to disease. All relapses and deaths were considered as events. Differences in RFS and OS were compared using Kaplan–Meier (K-M) curves.

The Goodman–Kruskal concordance index (c-index) is used as a metric to assess the models’ performance. It ranges between 0 and 1; however, a value of 0.5 indicates that a model does not perform better than random. The c-index is designed to estimate the concordance probability of independent and identically distributed data comparing the rankings of 2 independent survival times and hazard values [21,22]. Therefore, this index indicates the discriminatory properties and stratification accuracy. The precision of each risk classifier for RFS and OS (censored data) was evaluated using the Cox Proportional Hazards (PH) Model. The statistical analysis was based on Student’s t-test and the Mann–Whitney test for parametric and nonparametric continuous variables, respectively, and the chi-squared or Fisher’s exact test, as appropriate, for categorical variables. Statistical significance was considered when *p* < 0.05. Also, patients’ shifts between risk groups of different classification systems were illustrated by a Sankey diagram using Google Chart for developers (Google LLC, Menlo Park, CA, USA). Data were managed with an Excel database (Microsoft, Redmond, WA, USA) and statistical analyses were performed using R 4.0.3 software, available online at https://cran.r-project.org/ (accessed on 28 December 2021).

## 3. Results

### 3.1. Description of Clinical Characteristics

A total of 293 patients were included, with a median follow-up of 75 months. The clinicopathological characteristics of the entire cohort and their univariate analysis for RFS and OS are summarised in Table 1.

The majority of patients had tumours with endometrioid histological subtype (88.4%), low grade (80.2%) and FIGO stage Ia (69.3%). Lymphadenectomy was performed in 67.6% of patients (48.2% only pelvic; 19.4% pelvic and paraaortic). Adjuvant radiation therapy and chemotherapy were administered to 36.9% and 5.1% patients, respectively, but they did not show any significant impact in RFS or OS (data not shown). Relapse was identified in 43 (14.71%) patients, with a location pattern divided into locoregional (34.9%) and distant metastases (65.1%). Twenty-six (8.8%) deaths due to EC were recorded. All clinicopathological variables had a statistically significant correlation with RFS and OS (with the exception of LVSI in OS).

### 3.2. Prognosis Features and Accuracy of Stratification Tools

**Figure 1:**
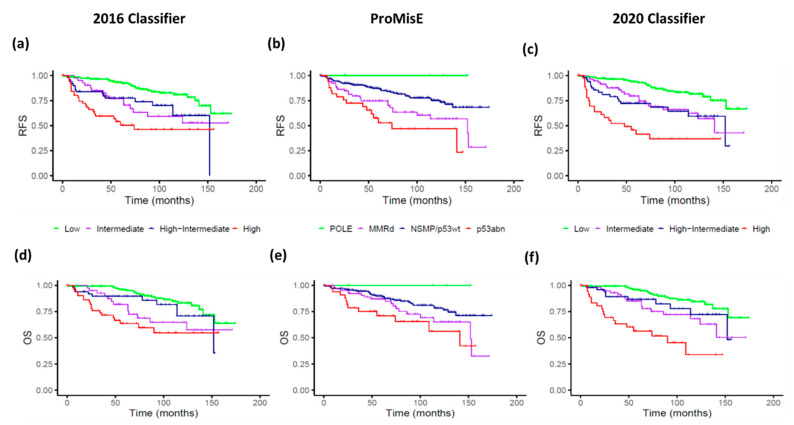
Relapse-free survival (RFS) and overall survival (OS) curves estimation by stratification prognosis tools. Upper row illustrates RFS for the 2016 Classifier (**a**), ProMisE (**b**), and 2020 Classifier (**c**). Lower row illustrates OS for the 2016 Classifier (**d**), ProMisE (**e**), and 2020 Classifier (**f**). The 2016 Classifier refers to the ESMO-ESGO-ESTRO classification, ProMisE to the Proactive Molecular Risk Classifier for Endometrial Cancer, and the 2020 Classifier to ESGO-ESTRO-ESP.

K-M curves for RFS and OS of each classifier are shown in Figure 1.

The distribution of prognosis risk groups, 5-year survival rate, Cox regression and c-index analysis for each classifier are detailed in Table 2.

Regarding the 2016 Classifier, the low-risk group is the most represented, accounting for half of the patients. The K-M curves showed a clear differentiation between low- and high-risk groups, with an early overlap of the intermediate groups’ curves.

The ProMisE classification found that p53/NSMP followed by MMRd groups represented the majority of cases. According to the selection of pathogenic variants proposed by León-Castillo et al. [18], in our series we identified five POLE patients that constitute two percent of total cases. Another seven patients presented additional alterations in *POLE* EDM, which were not used for classification purposes. The K-M curves confirmed that POLE and p53abn were the extreme prognosis groups. The MMRd group showed a poorer 5-year survival rate than p53wt/NSMP, but without significant differences.

**Figure 2:**
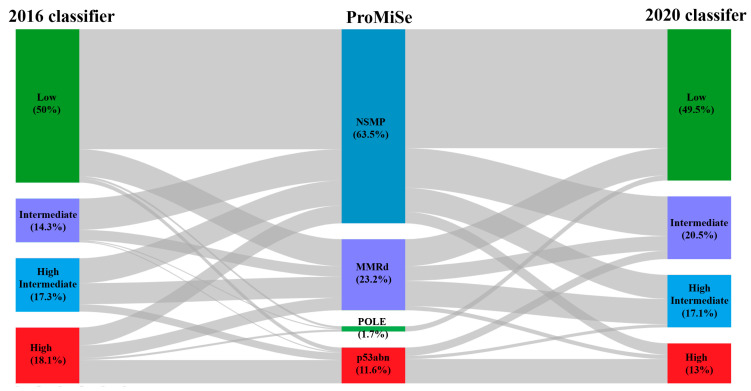
Sankey diagram. Risk groups are illustrated by coloured boxes, and percentage of cases within them are included for each classifier. Grey areas indicate case redistribution flow.

Lastly, regarding the 2020 Classifier, the low-risk group was the most frequent, with a similar proportion as that of the 2016 Classifier. However, there was a redistribution of the other three groups, with a decrease in the percentage of high-risk cases, and a redistribution of the intermediate and high-intermediate risk groups. Figure 2 illustrates shifts between the three stratification systems analysed.

Relapse survival analysis over intermediate and high-intermediate risk groups showed better differentiation between K-M curves but still narrow separation and late overlapping between these intermediate groups.

The Cox regression model for RFS found statistically significant differences for both the 2016 and 2020 Classifiers (*p* < 0.01), but not for ProMisE. Discriminative metrics in the entire cohort showed that the 2020 Classifier reached the highest c-index (0.78), closely followed by the 2016 Classifier (0.76). Despite the slight improvement in c-index value, when we look forward to the 5-year survival rates estimation, this showed that the redistribution among groups over the 2020 Classifier achieved a better RFS stratification compared to the 2016 Classifier (Table 2).

The Cox regression model was also performed for OS, finding again statistical significance for risk assessment in the 2016 and 2020 Classifiers: HR 1.53 (95% CI 1.25–1.87) and 1.79 (95% CI 1.44–2.23), respectively; *p* < 0.01 for both. In contrast, there was still an absence of significant differences for ProMisE (*p* = 0.57, for both outcomes).

### 3.3. Other Biomarker Assessments

The univariate statistics of other biomarkers for RFS and OS are provided in Table 3. ER and ECAD expression were the only biomarkers significantly correlated with a longer RFS and OS.

We also performed a subgroup analysis by histology and differentiation grade. Considering only the endometrioid histology subgroup, the *CTNNB1* mutation was associated with a significantly poorer RFS, whereas ER expression was correlated with a better OS and a trend towards a longer RFS (Supplementary Table S2). In the non-endometrioid subgroup, L1CAM expression had a trend to a longer RFS and ECAD to a longer OS (Supplementary Table S3). In the low-grade (histological differentiation grade 1 and 2) subgroup, there was a trend to a shorter RFS and OS with PTEN expression (Supplementary Table S4). None of the biomarkers showed a correlation with RFS or OS in the high-grade subgroup (Supplementary Table S5).

A descriptive analysis of these biomarkers regarding their distribution by the risk classifier categories is summarised in Supplementary Table S6.

**Figure 3:**
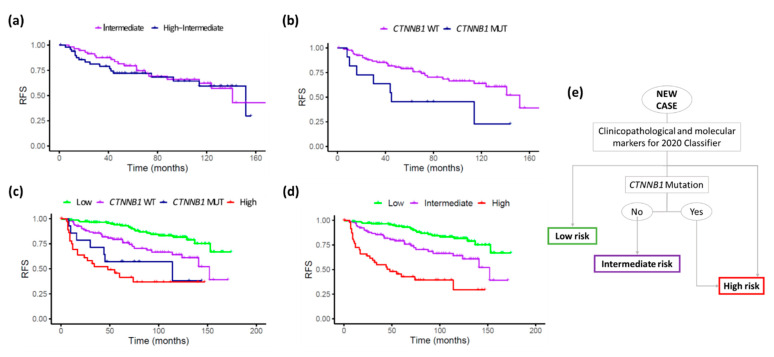
KM relapse-free survival plots for the new risk stratification proposal. (**a**) Intermediate and high-intermediate groups following the 2020 Classifier criteria; (**b**) merged intermediate groups stratified by *CTNNB1* status; (**c**) entire series, including high and low-risk groups based on 2020 Classifier criteria and intermediate groups stratified by *CTNNB1* status; and (**d**) entire series with the new proposal, stratified in 3 groups, by adding those with *CTNNB1* mutation to the high-risk group. (**e**) Decision tree based on this proposal.

As we explained before, our results showed that the 2020 Classifier was a slightly better stratification tool than the 2016 and ProMisE Classifiers in our series. However, the intermediate groups (intermediate and high-intermediate) still overlapped in RFS (Figure 1c and Figure 3a). Therefore, we merged these intermediate groups and performed a Cox regression analysis to explore the impact of the selected biomarkers (Supplementary Table S7). Among them, *CTNNB1* mutational status was the only one significantly associated to a shorter RFS (HR 2.62; 95% CI 1.14–6.02), and also showed a trend towards a worse OS (HR 2.17; 95% CI 0.81–5.78).

The K-M plots on the merged intermediate groups after categorization by *CTNNB1* mutation status showed an improved stratification (Figure 3b). Therefore, we substituted the two original intermediate 2020 Classifier groups for these new ones, while maintaining the original low- and high-risk groups (Figure 3c). Subsequently, we observed that patients with tumours harbouring the *CTNNB1* mutation showed a poor prognosis, with a similar RFS to the high-risk group (late curves overlapping). Thus, we proposed a novel stratification model consisting of three categories instead of four, by merging the 2020 Classifier high risk group with *CTNNB1* mutated tumours. The intermediate group was redefined as *CTNNB1* non-mutated cases from the previous intermediate risk groups (Figure 3d). A decision-tree model based on this proposal is shown in Figure 3e.

This new stratification system of three categories improved the c-index to 0.81 compared with 0.78 from the 2020 Classifier and reached statistically significant HR values for both the intermediate (2.47, *p* < 0.01) and the high-risk (7.10, *p* < 0.01) groups. Furthermore, it achieved statistically significant and clinically relevant differences in 5-year RFS: 93.9% for low risk, 79.1% for the intermediate merged group/*CTNNB1* wild type and 42.7% for the high-risk group (including patients from the merged intermediate groups with *CTNNB1* mutation).

## 4. Discussion

In this study, the three main risk classifiers described in the last decade (ESMO-ESGO-ESTRO 2016, ProMisE and ESGO-ESTRO-ESP 2020) were evaluated in a large early-stage EC cohort. The results showed that all of these classifiers differentiate RFS between high- and low-risk groups, but there was an overlap between the intermediate- and high-intermediate risk groups. Similar findings have been observed in other studies. For example, regarding the 2016 Classifier, two retrospective cohorts reported no differences between the intermediate and high-intermediate group, one of them with overlapping K-M OS curves [23,24]. In terms of the ProMisE Classifier, there are other publications that also showed no significant differences between the two intermediate molecular subtypes, although it performed well on the two extreme groups: the POLE group, with an excellent prognosis and a very low incidence of relapses, and the p53abn group, with the worst prognosis and a high risk of recurrence [25,26].

The distribution of cases by ProMisE groups in our series is lower for POLE, MMRd and NSMP than the originally described distribution. The main explanation for this is that TCGA groups may vary according to clinicopathological characteristics, as previously described [27,28]. Specifically, for the POLE group, it can also be explained because of technical modifications. In the ProMisE study, mutations were determined covering the EDM domain, and including all pathogenic variants within it. We have modified this classification for *POLE* status with the proposed list of mutations recently described by Leon-Castillo et al., which reduces the number of variants to take into account to 11 [18]. Different publications support overall that *POLE*-mutated cases have better prognosis outcomes, but in our knowledge, the consideration of isolate molecular features encourage a lack of information during prognosis stratification and needs more studies with homogeneity to clearly define this group [18,29,30,31].

The recently published 2020 Classifier has incorporated the molecular profile of the ProMisE classification into the prognostic stratification carried out in the 2016 Classifier, with the aim of improving its accuracy and thus making better therapeutic recommendations. In this new classification, stage I-II *POLE* mutated tumours are included in the low-risk group, for which adjuvant treatment is not recommended, whereas most of the p53abn tumours (except those without myometrial invasion) have been incorporated into the high-risk group, for which adjuvant chemotherapy is strongly recommended.

In this study, we have provided one of the first evaluations of this new risk classification in a cohort of patients and, to our knowledge, the first comparison of the three classifiers focused on early-stage EC. Two recent publications have evaluated the 2020 Classifier in two large patient cohorts, including those with advanced disease [32,33]. Similar to our results, Ortoft et al. described fewer patients allocated to the high-risk group using the 2020 Classifier and reported a poorer RFS for this group than that achieved with the 2016 Classifier [32]. These findings suggest that the 2020 Classifier achieves a better redistribution of the four risk groups that impact the 5-year survival rates. However, in terms of c-index values, we found only a slight improvement over the 2016 Classifier, associated to a small increase in the HR value. Furthermore, in our experience this classifier is still not good enough to separate the two intermediate groups, and following this classification, different adjuvant treatments would be recommended to patients with a similar prognosis (intermediate and high-intermediate groups). In the same way, Imboden et al. found significant differences in RFS using the 2020 Classifier, but with an overlap of K-M curves of both intermediate-risk groups [33]. These results reaffirm the unmet need for an accurate stratification system and motivate us to explore the potential of other biomarkers that could improve the current options.

To improve the precision of the 2020 Classifier, we focused on the molecular biomarkers previously explored in EC, with potential prognostic value but not yet included in the main risk classifiers. We first evaluated their association with prognosis in our entire cohort. Among them, only ER and ECAD showed a significant correlation with RFS and OS. These results are in agreement with previous publications [34,35]. There are several reports on HER2 amplification, specifically in non-endometrioid histologies and a subset of high-grade endometrioid tumours. We had almost no HER2 overexpression, so no correlations with the prognosis could be established [36]. Loss of ARID1A has been linked to shorter progression-free survival in EC, and loss of PTEN might be a good prognostic factor [37,38]. Our results are similar in terms of the positive proportion of cases for both biomarkers, but we did not find any statistical significance related to survival.

Among the remaining analysed markers, probably the most intriguing results concern L1CAM, which has frequently been associated with distant recurrence and OS. We have used a previously established cut-off for IHC to achieve the best correlation with prognosis [39]. Our results are similar regarding positivity rates to those published for the PORTEC-1 trial samples, but do not reach significance, probably because of the lower positivity of the marker and the smaller size of our cohort [40]. The other biomarker frequently associated with prognosis is *CTNNB1* [13,41]. In our cohort, it showed significance only when intermediate risk groups were merged, and for this reason it was subsequently considered for their inclusion in the risk classifier.

The impact of the *CTNNB1* mutation and other biomarkers (like POLE, MMRd, p53, L1CAM, or LVSI) prompted the design of the PORTEC-4 trial. In this phase III study, patients with high-intermediate risk EC are randomised between a standard arm with adjuvant vaginal brachytherapy and an experimental arm with adjuvant radiation therapy tailored by a molecular-integrated risk profile. In this trial, patients with p53wt/NSMP and no mutation in *CTNNB1* are considered to be in the same low-risk group as those with the *POLE* mutation [42]. However, in our study, patients initially classified in the intermediate or high-intermediate groups with no mutation in *CTNNB1* have a poorer prognosis than those of the low-risk group (which included patients with the *POLE* mutation).

The *CTNNB1* mutation leads to the overactivation of beta catenin, which results in the aberrant signalling of the Wnt pathway, contributing to tumour progression [43]. The poorer prognosis associated with the *CTNNB1* mutation in exon 3 has been shown in other studies, mainly in grade 1–2 endometrioid or NSMP cohorts [8,44], suggesting that this mutation is more likely to be functional, and not a passenger event [41]. Another study showed how the identification of *CTNNB1* alterations, along with *ARID1A* mutations, could represent an effective way to characterize the tumor aggressiveness of the heterogenous NSMP group [45]. However, although the ESGO-ESTRO-ESP 2020 guidelines mention that the *CTNNB1* mutation might be potentially useful in the group of low-grade p53wt/NSMP EC, they did not include it in the risk stratification proposal. In our study, the *CTNNB1* status was significantly associated with RFS in the intermediate and high-intermediate risk groups. Further, the *CTNNB1* mutational analysis over both intermediate groups could reallocate some patients into the high-risk group (those with the *CTNNB1* mutation), while the remaining patients would be considered within the intermediate-risk group. Moreover, by including the *CTNNB1* status in the 2020 Classifier, we simplified the four-group classification into three groups. Based on this proposal, adjuvant treatment recommendations could be made for each novel group; for example, patients allocated as intermediate or high-intermediate by the 2020 Classifier with the *CTNNB1* mutation can be considered for adjuvant chemotherapy.

The main limitation of our study is related to its retrospective design and the absence of a validation cohort. Therefore, our proposal of risk classifier needs to be validated in other external series, preferably from different countries and including a variety of ethnic groups, in order to confirm that the inclusion of *CTNNB1* status in the 2020 classifier improves its accuracy. Second, the study is based on TMA and not on whole tissue sections, which might not completely reflect the heterogeneity of some tumours. On the other hand, as strengths, the large number of patients with a long follow-up, and the high homogeneity of the series should be highlighted, given it encompasses only early stages (FIGO I-II). Furthermore, it is the first study to evaluate and compare the three most important risk classifiers in EC, including the recent ESGO-ESTRO-ESP Classification, focused on early-stage disease.

## 5. Conclusions

None of the main published risk classifiers developed in EC achieved a significant difference in RFS between their intermediate groups. The 2020 ESGO-ESTRO-ESP classification showed a slightly better discriminatory capacity than the other classifications. The incorporation of additional biomarkers, such as *CTNNB1*, into the 2020 Classifier could improve the accuracy of the stratification, especially in terms of redefining the intermediate prognostic groups. This proposal warrants validation in an external series, preferably from different countries and including a variety of ethnic groups.

## Figures and Tables

**Table 1:** Clinicopathological characteristics univariate analysis.

Variable	Descriptive	RFS		OS	
	*n* (%)	HR (95% CI)	*p* Value	HR (95% CI)	*p* Value
Age		1.67 (1.01–2.75)	0.04	1.90 (1.06–3.40)	0.03
<60 years	107 (36.5)				
>60 years	186 (63.5)				
Histological subtype		0.35 (0.19–0.62)	<0.01	0.40 (0.20–0.77)	<0.01
NEEC	34 (11.6)				
EEC	259 (88.4)				
Tumor grade		2.69 (1.65–4.37)	<0.01	2.86 (1.67–4.90)	<0.01
Low (G1 + G2)	235 (80.2)				
High (G3)	58 (19.8)				
Myometrial invasion		3.28 (1.43–7.54)	<0.01	3 (1.20–7.47)	0.02
No	58 (19.8)				
Yes	234 (79.9)				
NE	1 (0.3)				
LVSI		1.89 (1.13–3.14)	0.01	1.42 (0.76–2.62)	0.26
No	238 (81.2)				
Yes	53 (18)				
NE	2 (0.7)				
FIGO stage		2.11 (1.51–2.95)	<0.01	1.88 (1.28–2.76)	<0.01
Ia	203 (69.3)				
Ib	73 (24.9)				
II	17 (5.8)				

EEC: Endometrioid endometrial carcinoma; NEEC: Non-endometrioid endometrial carcinoma; NE: No evaluable; LVSI: Lymph-vascular space invasion; FIGO: International Federation of Gynecology and Obstetrics; HR: Hazard ratio. 95% CI: 95% Confidence interval; RFS: Relapse-free survival; OS: Overall survival.

**Table 2:** Risk stratification tools accuracy comparison by relapse-free survival.

Classifier	Descriptive		RFS		
	*n* (%)	5-Year Rate (%)	HR (95% CI)	*p*-Value	c-Index
2016 Classifier			1.62 (1.35–1.95)	<0.01	0.76
Low	147 (50)	93.2			
Intermediate	42 (14.3)	76.9			
High-intermediate	51 (17.3)	77.5			
High	53(18.1)	50.4			
ProMisE			1.19 (0.81–1.74)	0.37	0.53
POLE	5 (1.7)	100			
MMRd	68 (23.2)	74.7			
p53wt/NSMP	186 (63.5)	87.3			
p53abn	34 (11.6)	52.8			
2020 Classifier			1.85 (1.53–2.25)	<0.01	0.78
Low	145 (49.5)	93.9			
Intermediate	60 (20.5)	79.5			
High-intermediate	50 (17.1)	72.2			
High	38 (13)	41.5			

RFS: Relapse-free survival; POLE: Polymerase ε exonuclease domain mutation; MMRd: Mismatch repair deficiency. P53wt/NSMP: P53 wild type/Non-specific molecular profile; p53abn: p53 aberrant; HR: Hazard ratio; 95% CI: 95% Confidence interval.

**Table 3:** Univariate biomarker analysis for relapse-free survival and overall survival.

Variable	Descriptive	RFS		OS	
	*n* (%)	HR (95% CI)	*p*-Value	HR (95% CI)	*p*-Value
ER		0.39 (0.23–0.67)	<0.01	0.34 (0.19–0.62)	<0.01
Negative	39 (13.3)				
Positive	244 (83.3)				
NE	10 (3.4)				
PR		0.61 (0.35–1.08)	0.09	0.55 (0.30–1.02)	0.06
Negative	44 (15)				
Positive	235 (80.2)				
NE	14 (4.8)				
ECAD		0.57 (0.33–0.98)	0.04	0.47 (0.26–0.85)	0.01
Negative	46 (15.7)				
Positive	234 (79.9)				
NE	13 (4.4)				
HER2		1.17 (0.16–8.40)	0.88	NE	NE
Negative	289 (98.6)				
Positive	3 (1)				
NE	1 (0.3)				
ARID1A		0.87 (0.50–1.51)	0.62	0.94 (0.51–1.73)	0.84
Negative	219 (74.7)				
Positive	61 (20.8)				
NE	13 (4.4)				
PTEN		1.27 (0.80–2.01)	0.31	1.24 (0.73–2.09)	0.42
Negative	193 (65.9)				
Positive	94 (32.1)				
NE	6 (2.0)				
L1CAM		1.30 (0.62–2.73)	0.48	1.34 (0.57–3.14)	0.50
Negative	247 (84.3)				
Positive	32 (10.9)				
NE	14 (4.8)				
CTNNB1		1.58 (0.79–3.17)	0.20	1.24 (0.53–2.89)	0.62
Non mutated	249 (85)				
Mutated	23 (7.8)				
NE	21 (7.2)				

NE: Not evaluable; HR: Hazard ratio; 95% CI: 95% Confidence interval; RFS: Relapse-free survival; OS: Overall survival.

## Data Availability

Data are available on reasonable request from authors.

## Supplementary Materials

The following supporting information can be downloaded at: https://www.mdpi.com/article/10.3390/cancers14040912/s1. Supplementary Table S1. Immunohistochemistry evaluation. Supplementary Table S2. Univariate biomarker analysis in endometrioid subtype cohort. Supplementary Table S3. Univariate biomarker analysis in non endometrioid subtype cohort. Supplementary Table S4. Univariate biomarker analysis in low-grade cohort. Supplementary Table S5. Univariate biomarker analysis in high-grade cohort. Supplementary Table S6. Biomarker distribution among risk classifiers. Supplementary Table S7. Univariate biomarker analysis over 2020 Classifier intermediate groups merged cohort.
