# Supplementary material for: Prognosis Stratification Tools in Early-Stage Endometrial Cancer: Could We Improve Their Accuracy?
Source: Cancers (Basel). 2022 Feb 12;14(4):912. doi: 10.3390/cancers14040912 (PMC8869938; doi:10.3390/cancers14040912)
Supplement: Supplementary file 1 [file cancers-14-00912-s001.zip › cancers-1560558-supplementary.pdf]

cancers

MDPI

Supplementary Material: Prognosis Stratification Tools in Early-Stage Endometrial Cancer: Could We Improve Their Accuracy?

Jorge Luis Ramon-Patino, Ignacio Ruz-Caracuel, Victoria Heredia-Soto, Luis Eduardo Garcia de la Calle, Bulat Zagidullin, Yinyin Wang, Alberto Berjon, Alvaro Lopez-Janeiro, Maria Miguel, Javier Escudero, Alejandro Gallego, Beatriz Castelo, Laura Yebenes, Alicia Hernandez, Jaime Feliu, Alberto Pelaez-García, Jing Tang, David Hardisson, Marta Mendiola and Andres Redondo

Supplementary Table S1. Immunohistochemistry evaluation.

Biomarker	Antibody characteristics (clone. ref)	Positivity cut-off
ER	EP1 (#IR084)[1]	1%
PR	PgR1294 (#M3568)[1]	1%
ECAD	NCH-38 (#M3612)[1]	Preserved versus lost expression
HER2	DG44 (Herceptest)[1]	0 to 1+: HER2-negative<br>3+: HER2-positive<br>2+: equivocal*
ARID1A	EPR13501 (#182560)[2]	1%
PTEN	6H2.1 (M3627)[1]	1%
L1CAM	UJ127.11 (ab2014)[1]	10%

[1]Dako-Agilent; [2]Abcam. *Fluorescence in situ hybridization (FISH) method was performed for reclassification.

Supplementary Table S2. Univariate biomarker analysis in endometrioid subtype cohort.

Variable	Descriptive	RFS		OS	
	n (%)	HR (95%CI)	p-value	HR (95%CI)	*p*-value
ER					
Negative	14 (5.4)				
Positive	236 (91.1)	0.45 (0.19 – 1.05)	0.06	0.37 (0.16 – 0.88)	0.02
NE	9 (3.5)				
PR					
Negative	20 (7.7)				
Positive	226 (87.3)	0.97 (0.39 – 2.44)	0.95	0.79 (0.31 – 1.99)	0.61
NE	13 (5.0)				
ECAD					
Negative	35 (13.5)				
Positive	216 (83.4)	0.77 (0.39 – 1.52)	0.45	0.65 (0.31 – 1.33)	0.24
NE	8 (3.1)				
HER2					
Negative	258 (99.6)	NE	NE	NE	NE
Positive	1 (0.4)				
NE	–				
ARID1A					
Negative	191 (73.7)				
Positive	55 (21.2)	0.76 (0.41 – 1.43)	0.39	0.83 (0.41 – 1.65)	0.59
NE	13 (5.0)				

PTEN<br>Negative<br>Positive<br>NE	174 (67.2)<br>80 (30.9)<br>5 (1.9)	1.44 (0.87 – 2.40)	0.16	1.47 (0.83 – 2.61)	0.19
L1CAM<br>Negative<br>Positive<br>NE	235 (90.7)<br>12 (4.6)<br>12 (4.6)	1.28 (0.40 – 4.09)	0.68	1.71 (0.53 – 5.51)	0.37
CTNNB1<br>Non mutated<br>Mutated<br>NE	224 (86.5)<br>20 (7.7)<br>15 (5.8)	2.11 (1.04 – 4.27)	0.04	1.51 (0.64 – 3.56)	0.34

NE: Not evaluable; HR: Hazard ratio; 95%CI: 95% Confidence interval; RFS: Relapse-free survival; OS: Overall survival.

Supplementary Table S3. Univariate biomarker analysis in non endometrioid subtype cohort.

Variable	Descriptive	RFS		OS	
	n (%)	HR (95%CI)	p-value	HR (95%CI)	p-value
ER<br>Negative<br>Positive<br>NE	25 (73.5)<br>8 (23.5)<br>1 (2.9)	0.89 (0.25 – 3.20)	0.86	0.66 (0.14 – 3.08)	0.60
PR<br>Negative<br>Positive<br>NE	24 (70.6)<br>9 (26.5)<br>1 (2.9)	1.15 (0.36 – 3.70)	0.81	0.96 (0.25 – 3.62)	0.95
ECAD<br>Negative<br>Positive<br>NE	11 (32.4)<br>18 (52.9)<br>5 (14.7)	0.45 (0.15 – 1.36)	0.16	0.25 (0.06 – 0.99)	0.05
HER2<br>Negative<br>Positive<br>NE	31 (91.2)<br>2 (5.9)<br>1 (2.9)	0.62 (0.08 – 4.84)	0.65	NE	NE
ARID1A<br>Negative<br>Positive<br>NE	28 (82.4)<br>6 (17.6)<br>-	1.53 (0.48 – 4.84)	0.47	1.66 (0.44 – 6.32)	0.46
PTEN<br>Negative<br>Positive<br>NE	19 (55.9)<br>14 (41.2)<br>1 (2.9)	0.46 (0.16 – 1.37)	0.16	0.38 (0.10 – 1.44)	0.15
L1CAM<br>Negative<br>Positive<br>NE	12 (35.3)<br>20 (58.8)<br>2 (5.9)	0.35 (0.11 – 1.08)	0.07	0.31 (0.08 – 1.25)	0.10
CTNNB1<br>Non mutated<br>Mutated<br>NE	25 (73.5)<br>3 (8.8)<br>6 (17.6)	NE	NE	NE	NE

NE: Not evaluable; HR: Hazard ratio; 95%CI: 95% Confidence interval; RFS: Relapse-free survival; OS: Overall survival.

Supplementary Table S4. Univariate biomarker analysis in low-grade cohort.

Variable	Descriptive	RFS		OS	
	n (%)	HR (95%CI)	p-value	HR (95%CI)	p-value
ER					
Negative	3 (1.3)				
Positive	225 (95.7)	0.72 (0.10 – 5.24)	0.75	0.54 (0.07 – 3.91)	0.54
NE	7 (3.0)				
PR					
Negative	11 (4.7)				
Positive	213 (90.6)	2.9 (0.40 – 21.03)	0.29	2.14 (0.29 – 15.65)	0.45
NE	11 (4.7)				
ECAD					
Negative	27 (11.5)				
Positive	202 (86.0)	0.61 (0.29 – 1.30)	0.20	0.49 (0.22 – 1.11)	0.09
NE	6 (2.6)				
HER2					
Negative	235 (100)				
Positive	0	NE	NE	NE	NE
NE	-				
ARID1A					
Negative	176 (74.9)				
Positive	49 (20.9)	0.74 (0.37 – 1.47)	0.39	0.80 (0.37 – 1.72)	0.56
NE	10 (4.3)				
PTEN					
Negative	158 (67.2)				
Positive	73 (31.1)	1.71 (0.99 – 2.94)	0.05	1.79 (0.96 – 3.35)	0.07
NE	4 (1.7)				
L1CAM					
Negative	220 (93.6)				
Positive	6 (2.6)	0.76 (0.11 – 5.52)	0.79	1.03 (0.14 – 7.48)	0.98
NE	9 (3.8)				
CTNNB1					
Non mutated	204 (86.8)				
Mutated	17 (7.2)	1.94 (0.87 – 4.29)	0.10	1.21 (0.43 – 3.39)	0.72
NE	14 (6.0)				

NE: Not evaluable; HR: Hazard ratio; 95%CI: 95% Confidence interval; RFS: Relapse-free survival; OS: Overall survival.

Supplementary Table S5. Univariate biomarker analysis in high-grade cohort.

Variable	Descriptive	RFS		OS	
	n (%)	HR (95%CI)	p-value	HR (95%CI)	p-value
ER					
Negative	36 (62.1)				
Positive	19 (32.8)	0.69 (0.27 – 1.76)	0.44	0.66 (0.24 – 1.84)	0.43
NE	3 (5.2)				
PR					
Negative	33 (56.9)				
Positive	22 (37.9)	0.89 (0.37 – 2.14)	0.80	0.94 (0.37 – 2.40)	0.89

NE	3 (5.2)				
ECAD					
Negative	19 (32.8)				
Positive	32 (55.2)	0.83 (0.35 – 1.97)	0.67	0.68 (0.26 – 1.77)	0.43
NE	7 (12.1)				
HER2					
Negative	54 (93.1)				
Positive	3 (5.2)	0.49 (0.07 – 3.67)	0.49	NE	NE
NE	1 (1.7)				
ARID1A					
Negative	43 (74.1)				
Positive	12 (20.7)	1.12 (0.44 – 2.85)	0.82	1.19 (0.43 – 3.33)	0.74
NE	3 (5.2)				
PTEN					
Negative	35 (60.3)				
Positive	21 (36.2)	0.52 (0.21 – 1.25)	0.14	0.44 (0.16 – 1.22)	0.12
NE	2 (3.4)				
L1CAM					
Negative	27 (46.6)				
Positive	26 (44.8)	0.55 (0.22 – 1.38)	0.20	0.51 (0.18 – 1.47)	0.21
NE	5 (8.6)				
CTNNB1					
Non mutated	45 (77.6)				
Mutated	6 (10.3)	0.76 (0.18 – 3.28)	0.71	1.09 (0.25 – 4.78)	0.91
NE	7 (12.1)				

NE: Not evaluable; HR: Hazard ratio; 95%CI: 95% Confidence interval; RFS: Relapse-free survival; OS: Overall survival.

Supplementary Table S6. Biomarker distribution among risk classifiers.

**2016 Classifier**	**Low**	**Intermediate**	**High-intermediate**	**High**
	**n (%)**	**n (%)**	**n (%)**	**n (%)**
ER	141 (95.9)	41 (97.6)	43 (84.3)	19 (35.8)
PR	131 (89.1)	41 (97.6)	41 (80.4)	22 (41.5)
ECAD	132 (89.8)	35 (83.3)	41 (80.4)	26 (49.1)
HER2	0	0	1 (2.0)	2 (3.8)
ARID1A	28 (19.0)	6 (14.3)	15 (29.4)	12 (22.6)
PTEN	50 (34.0)	14 (33.3)	13 (25.5)	17 (32.1)
L1CAM	3 (2.0)	1 (2.4)	7 (13.7)	21 (39.6)
CTNNB1	9 (6.1)	4 (9.5)	4 (7.8)	6 (11.3)
**ProMisE**	**POLE**	**MMRd**	**p53wt/NSMP**	**p53abn**
	**n (%)**	**n (%)**	**n (%)**	**n (%)**
ER	3 (60.0)	59 (86.8)	165 (88.7)	17 (50.0)
PR	3 (60.0)	52 (76.5)	162 (87.1)	18 (52.9)
ECAD	3 (60.0)	57 (83.8)	153 (82.3)	21 (61.8)
HER2	0	0	1 (0.5)	2 (5.9)
ARID1A	0	21 (30.9)	35 (18.8)	5 (14.7)
PTEN	2 (40.0)	13 (19.1)	66 (35.5)	13 (38.2)
L1CAM	0	5 (7.4)	10 (5.4)	17 (50.0)
CTNNB1	1 (20.0)	2 (2.9)	18 (9.7)	2 (5.9)
**2020 Classifier**	**Low**	**Intermediate**	**High-intermediate**	**High**

	n (%)	n (%)	n (%)	n (%)
ER	137 (94.5)	50 (83.3)	42 (84.0)	15 (39.5)
PR	127 (87.6)	50 (83.3)	41 (82.0)	17 (44.7)
ECAD	129 (89.0)	50 (83.3)	36 (72.0)	19 (50.0)
HER2	0	1 (1.7)	0	2 (5.3)
ARID1A	28 (19.3)	7 (11.7)	18 (36.0)	8 (21.1)
PTEN	52 (35.9)	18 (30.0)	9 (18.0)	15 (39.5)
L1CAM	4 (2.8)	10 (16.7)	3 (6.0)	15 (39.5)
CTNNB1	9 (6.2)	7 (11.7)	4 (8.0)	3 (7.9)

POLE: Polymerase ε exonuclease domain mutation; MMRd: Mismatch repair deficiency; p53wt/NSMP: p53 wild type/Non-specific molecular profile; p53abn: p53 aberrant.

Supplementary Table S7. Univariate biomarker analysis over 2020 Classifier intermediate groups merged cohort.

Variable	Descriptive	RFS		OS	
	n (%)	HR (95%CI)	p-value	HR (95%CI)	p-value
ER					
Negative	13 (11.8)				
Positive	92 (83.6)	2.41 (0.56 – 10.32)	0.24	1.64 (0.38 – 7.10)	0.51
NE	5 (4.5)				
PR					
Negative	15 (13.6)				
Positive	91 (82.7)	3.04 (0.72 – 12.89)	0.13	2.09 (0.49 – 8.99)	0.32
NE	4 (3.6)				
ECAD					
Negative	20 (18.2)				
Positive	86 (78.2)	0.78 (0.34 – 1.79)	0.56	0.57 (0.23 – 1.42)	0.23
NE	4 (3.6)				
HER2					
Negative	109 (99.1)				
Positive	1 (0.9)	NE	NE	NE	NE
NE	0 (0)				
ARID1A					
Negative	81 (73.6)				
Positive	25 (22.7)	0.51 (0.20 – 1.32)	0.17	0.45 (0.14 – 1.52)	0.20
NE	4 (3.6)				
PTEN					
Negative	81 (73.6)				
Positive	27 (24.5)	1.21 (0.59 - 2.49)	0.60	1.59 (0.70 – 3.59)	0.26
NE	2 (1.8)				
L1CAM					
Negative	94 (85.5)				
Positive	13 (11.8)	0.23 (0.03 – 1.66)	0.14	0.37 (0.05 – 2.78)	0.34
NE	3 (2.7)				
CTNNB1					
Non mutated	95 (86.4)				
Mutated	11 (10.0)	2.62 (1.14 – 6.02)	0.02	2.17 (0.81 – 5.78)	0.12
NE	4 (3.6)				

NE: Not evaluable; HR: Hazard ratio; 95%CI: 95% Confidence interval; RFS: Relapse-free survival; OS: Overall survival.
